# Comprehensive Comparisons of Family Health Between Families With One Immigrant Parent and Native Families in Taiwan: Nationwide Population-Based Cohort Study

**DOI:** 10.2196/33624

**Published:** 2022-12-19

**Authors:** Yi-Lung Chen, Hsing-Ying Ho

**Affiliations:** 1 Department of Healthcare Administration Asia University Taichung Taiwan; 2 Department of Psychology Asia University Taichung Taiwan

**Keywords:** international marriage immigrant family, family health, physical health, mental health, mortality, reduced inequalities, good health and well-being

## Abstract

**Background:**

Mothers and children in families with one immigrant parent have been reported to be healthier than those in native families; however, the health of the fathers in these families has rarely been discussed in literature.

**Objective:**

We aimed to comprehensively compare the health of all the family members between families with one immigrant parent (native fathers, immigrant mothers, and their children) and native families (native fathers, native mothers, and their children).

**Methods:**

We conducted a cohort study by using the Taiwan Maternal and Child Health Database to recruit live-born children and their parents from 2004 to 2016. Overall, we identified 90,670 fathers, 91,270 mothers, and 132,457 children in families with one immigrant parent and 1,666,775 fathers, 1,734,104 mothers, and 2,637,191 children in native families and followed up with them from 2004 to 2017. The outcomes comprised common physical and mental disorders, catastrophic illnesses, mortality, and child adversities and accidents. The covariates comprised the child’s year of birth, parental age, low-income status, and physical or mental disorder status. Logistic regression was performed to compare the risks of the outcomes between families with one immigrant parent and native families.

**Results:**

The parents in families with one immigrant parent were more likely to be of low-income status and were older than the parents in native families. After adjusting for the covariates, fathers in families with one immigrant parent were found to have higher risks of physical and mental disorders, catastrophic illness, and mortality than fathers in native families. Conversely, mothers in families with one immigrant parent had lower risks of physical and mental disorders, catastrophic illness, and mortality than mothers in native families. Finally, the children in families with one immigrant parent generally had better physical and mental health but higher risks for leukemia, liver diseases, autism spectrum disorder, and road traffic accidents than children in native families.

**Conclusions:**

The health status of the members of families with one immigrant parent was nonhomogeneous, and the poorer general health of fathers in such families suggests health inequalities in families with one immigrant parent.

## Introduction

Currently, international migration is a common globalization phenomenon, and migrants compose an important part of the population in many countries. The number of international migrants reached 272 million worldwide in 2019 [1]. Marriage is one of the main causes of migration, especially in Asian countries [1] such as Japan, South Korea [2], Singapore [3], and Taiwan [4]. Individuals often migrate to wealthier neighboring countries to improve their living conditions [1]. In Taiwan, 95.6% of the naturalization applications from 1993 to 2019 were due to international marriage [5], which is defined as a marriage formed between 2 individuals from different countries of origin, resulting in 565,000 married migrants with a temporary or permanent residence permit as of 2019 [6]. Most married immigrants in Taiwan are females, and most of them migrated from East or Southeast Asian countries [4]. International marriage contributed to 9.1% of the overall marriages in Taiwan from 1998 to 2020 [4]. The newborns of married immigrants comprised 8.6% of the overall newborns in Taiwan from 1998 to 2020 [7]. These findings indicate that families with one immigrant parent have become an important part of the population in Taiwan, and this study focused on families with one immigrant parent (families consisting of a native father, an immigrant mother, and a child) and native families (families consisting of a native father, a native mother, and a child).

The literature is not in agreement regarding the differences in terms of general health between married female immigrants and married native women, and 2 different theories have been proposed to explain these inconsistent findings [8,9]. The first theory is the healthy immigrant effect, which asserts that immigrants generally have better health because healthier people are more likely to immigrate to seek a better life, and medical examinations required by immigration authorities in host countries may also prevent less healthy individuals from immigrating [8]. The healthy immigrant effect has been supported among married female immigrants in Taiwan [10]. Furthermore, although married female immigrants reported higher acculturative stress and lower spousal support, they reported fewer depressive symptoms than native women [11]. Another study indicated that married female immigrants had a better quality of life, fewer stressful life events, and a lower prevalence of major depressive disorder than married native women [12]. Moreover, fewer married female immigrants reported prenatal and postpartum depression and physical disorders than married native women [13]. The other theory is the salmon bias effect, which proposes that sick or older immigrants return to their countries of origin [14]. Thus, immigrants may not be truly healthy, and the disease and mortality of immigrants may be underestimated and that is why they return to their countries of origin. Some studies have partially supported the salmon bias effect in married female immigrants [10,14-16]. Specifically, married female immigrants were reported to be more likely to experience physical and mental disorders than native populations in Asian countries [10,15]. Furthermore, depression was one of the main concerns among mental disorders; married female immigrants reported having a higher prevalence of depression during the antenatal (31.8% vs 18.6%, respectively) [16] and postpartum period (41.1% vs 8.4%, respectively) [17,18] than native women. The higher risk of anxiety was another concern [19]. With regard to physical disorders, the risk of viral hepatitis in married female immigrants was higher than that in native women [20]. However, unlike married female immigrants, their native male spouses receive much less attention, and the difference in the general health between the native male spouses of native women and the native male spouses of married female immigrants remains unclear.

One evident feature of the native male spouses of married female immigrants is the low socioeconomic status (SES) [21,22], including older age [17,23], low education levels [24,25], employment in unskilled labor positions [26], and low income [25], thus hindering native men from getting married to native women and having possibly poorer health. Marriage migration in Taiwan originated in the 1960s, when retired veterans had difficulties getting married, which resulted in the development of international marriage brokerage agencies. Furthermore, by 1990, both the out-migration of industries and the import of foreign labor had a great impact on the employment of men in unskilled labor positions in Taiwan. In addition, the education levels of Taiwanese women increased in the 1990s. These situations made spouse selection difficult for men with low education levels and partially increased the average age of marriage for Taiwanese individuals [27]. As a result, these Taiwanese men with low SES tended to utilize international marriage brokerage agencies. In addition, the low SES of these native male spouses may be related to negative impacts on their health. Although several types of health status have been reported in native male spouses, including mental or physical disabilities [25,28], chronic diseases, serious illnesses [25], and general health issues [25,29], significant limitations existed in these studies. First, the studies were small-scale cross-sectional studies with sample sizes ranging from 140 to 1827 participants. Second, these studies only reported the prevalence of diseases, and a comparison with native male spouses who married native women is lacking. Third, because these studies were based on informant reports and not self-reports, the reliability and validity of the studies may be limited. Therefore, more comprehensive and large-scale studies with direct information from native male spouses of female immigrants are warranted for a better understanding of their health.

The mental health of children of married female immigrants has been reported to be generally worser than the mental health of children of native women, while comparison studies on physical health are relatively limited. Specifically, for mental health, more externalizing (eg, delinquent behavior) [19,23,30] and internalizing (eg, anxiety, depression) behavioral problems [19,30] were observed in the children of married female immigrants, although the results varied in school and family settings [30]. Furthermore, the depression levels of the children of married female immigrants were more likely to be affected by family factors [31]. In terms of physical health, newborns of married female immigrants had a lower risk of neonatal mortality than newborns of native women after adjusting for demographic confounders [32].

There are debatable findings on child adversity between children of native families and those of immigrant families. Some studies have shown that children of immigrant families experience a higher rate of maltreatment and domestic violence [33] and road traffic accidents [34], possibly as a result of low SES [33]. However, mental and physical disorders and child adversity have not been examined in Taiwan, and a comprehensive comparison of mental and physical disorders, adversity, and accidents between families with one immigrant parent and native families is lacking.

The aim of this study was to examine the family health between families with one immigrant parent and native families, and this analysis included the health of all the family members, that is, mothers (married female immigrants or native women), native fathers, and children. To address the aforementioned research gaps such as the small-scale cross-sectional study designs and comparisons of specific diseases, this study used a nationwide population-based cohort database to comprehensively compare the health of the family members between families with one immigrant parent and native families. We compared the risks of common physical and mental disorders, catastrophic illnesses, and mortality between all members of families with one immigrant parent and native families and the risks of domestic violence, maltreatment, sexual assault, and road traffic accidents between children of families with one immigrant parent and those of native families.

## Methods

### Population

The population data were derived from the Taiwan National Health Insurance Research Database, a medical claims database that includes data on all the medical visits for ambulatory care, emergency care, and hospitalization, which is compulsory social insurance for citizens, immigrants, foreign workers, and foreign students. Up to 99.9% of Taiwan’s population is enrolled in this database [35]. We used the Maternal and Child Health Database from the Taiwan National Health Insurance Research Database to extract complete information of live-born children regarding gestational age at birth and the identities of their parents. The Maternal and Child Health Database includes 99.78% of all births nationwide from 2004 to 2016 in Taiwan [36], which was followed up to 2017.

### Ethics Approval

This study was approved by the Research Ethics Committee of the China Medical University and Hospital (approval: CMUH108-REC1-142).

### Measures

#### Exposure

The exposure in this study was international married immigrant status. We used the record in the Taiwan Birth Certificate Registration to identify the nationality of the participants, and because the Taiwan Birth Certificate Registration data set from the Taiwan National Health Insurance Research Database contains only data of the mothers, a linkage to the Taiwan Maternal and Child Health Database was made to obtain the complete data of the children and their fathers. The inclusion criteria in this study were as follows: (1) live-born children included in the Taiwan Birth Certificate Registration and (2) no missing data of the children and their fathers and mothers. One exclusion criterion is that we excluded married female immigrants from South Korea, Japan, and western countries because the development index in these countries was close to or higher than that in Taiwan, and we restricted families with one immigrant parent to those in East or Southeast Asian countries, which were defined as China, Vietnam, Indonesia, Thailand, the Philippines, Malaysia, Myanmar, and Cambodia. The exposure group included family members from families with one immigrant parent (native fathers, immigrant mothers, and their children), whereas the nonexposure group included native families (native fathers, native mothers, and their children). A comparison was made separately for individual family members between families with one immigrant parent and native families.

#### Outcome

The outcomes in this study comprised common physical and mental disorders, Charlson comorbidity index, catastrophic illnesses, mortality, adversities, and accidents. The Charlson comorbidity index was originally designed as a measure to examine the risk of 1-year mortality from comorbid diseases in a longitudinal study of general hospital patients by taking the seriousness of comorbid diseases into account and weighting them to calculate a comorbidity score [37]. If participants have 1 of the 19 diseases, they receive a corresponding weight score. The assigned weights for diseases were as follows: 1 for myocardial infarction, congestive heart failure, peripheral vascular disease, cerebrovascular disease, dementia, chronic pulmonary disease, connective tissue disease, ulcer disease, mild liver disease, and diabetes; 2 for diabetes with end-organ damage, hemiplegia, moderate or severe renal disease, any tumor, leukemia, and lymphoma; 3 for moderate or severe liver disease; and 6 for metastatic solid tumor and AIDS. The potential range of the Charlson comorbidity index is from 0 to 37, with a higher score indicating worse comorbidity. It was subsequently adapted for use as an index of general health [38]. The Charlson comorbidity index and common mental disorders were used to represent physical health and mental health in this study and were determined based on the International Classification of Diseases, Ninth Revision and Tenth Revision codes in the Taiwan National Health Insurance Research Database. We excluded some uncommon mental disorders specific to children and adults because some disorders are differentially prevalent in different age groups. For example, regarding physical health, dementia is included in the Charlson comorbidity index, but dementia is not diagnosed in children, whereas for mental health, conduct disorder or oppositional defiant disorder (CD/ODD) and tic disorders are childhood mental disorders, and when individuals with conduct disorder reach adulthood, their symptoms may be exhibited as antisocial personality disorder [39]. Antisocial personality disorder is usually underdiagnosed and undertreated, and symptoms of tic disorders are usually relieved in adulthood. Specifically, for fathers and mothers, we used all disorders in the Charlson comorbidity index [40] and common mental disorders in adulthood (ie, autism spectrum disorder [ASD], attention deficit and hyperactivity disorder [ADHD], anxiety disorders, major depressive disorder, bipolar disorder, and schizophrenia); for children, we excluded myocardial infarction and dementia when using Charlson comorbidity index, and we included 2 additional childhood disorders (ie, tic disorder and CD/ODD). Participants were considered to have diseases if they received at least one inpatient diagnosis or more than 2 outpatient diagnoses from 2004 to 2017. Catastrophic illness was determined from the Registry for Catastrophic Illness Patients database, and the categories of diseases for catastrophic illnesses is listed in Table S1 of Multimedia Appendix 1. Mortality was determined by the Cause of Death Data. Child adversities and accidents were defined as the experience of domestic violence, maltreatment, sexual assault, and road traffic accidents and were extracted from the Family Violence Data and the Reported Data of Protection of Child and Youths, the Reported Data of Sexual Assault, and the Traffic Accident Data. Detailed information on these data sets is available in Multimedia Appendix 1.

#### Covariates

For the analysis of the physical and mental health of the parents, we controlled for age, low-income status, and geographical location (ie, northern, central, southern, and eastern Taiwan). The definition of the geographical location was based on the National Development Council [41]. For the analysis of physical and mental health in children, to control for hereditary factors, we examined the children’s physical and mental health and further controlled for their parents’ physical health (Charlson comorbidity index) and mental health (schizophrenia, bipolar disorder, ASD, and ADHD), as well as age, sex and low-income status.

### Statistical Analysis

SAS version 9.4 (SAS Institute Inc) was used for data management and analysis. Descriptive statistics were applied to indicate the frequency with percentage for categorical variables (ie, sex, low-income status, physical and mental disorders, mortality, and child adversities and accidents) and the mean with SD for continuous variables (ie, age and Charlson comorbidity index score). We used logistic regression for binary outcome variables (ie, physical and mental disorders) and linear regression for continuous outcome variables (ie, Charlson comorbidity index score) to compare the sociodemographic variables and risks of family health among fathers, mothers, and children in families with one immigrant parent and those in native families. For parents, we first performed an unadjusted analysis to compare the sociodemographic variables between families with one immigrant parent and native families and reported crude odds ratios or regression coefficients and 95% CIs. Furthermore, we performed an unadjusted analysis to examine family health while controlling for the sociodemographic variables.

Since the mothers in families with one immigrant parent did not have data on the health care utilization before their immigration unlike the mothers in native families, we performed a sensitivity analysis to restrict a similar period of health care utilization (the start date of health care utilization was the delivery date of their first child) as the native women. Furthermore, we compared the health between the fathers and mothers stratified by families with one immigrant parent and native families by using a similar analysis, and a moderation analysis was performed to examine whether the health between fathers and mothers differed between families with one immigrant parent and native families.

A similar analytical strategy was performed for between the children of families with one immigrant parent and those of native families. Furthermore, in the adjusted model, we first included parental age as a covariate, and we further controlled for the physical (ie, Charlson comorbidity index score) and mental health (ie, ASD, ADHD, and schizophrenia for mental disorders) of the parents separately when we examined the physical and mental health between children of families with one immigrant parent and those of native families. Finally, we further examined the risks of adversity and accidents between them.

## Results

Table 1 summarizes the sociodemographic data and the general health of the parents in families with one immigrant parent and those of parents in native families. We included 90,670 fathers, 91,270 mothers, and 132,457 children from families with one immigrant parent and 1,666,775 fathers, 1,734,104 mothers, and 2,637,191 children from native families over a period of 12 years. The fathers (age, 44.8 years vs 40.2 years, respectively) and mothers (age, 35.2 years vs 34.4 years, respectively) in families with one immigrant parent were older than the fathers and mothers in native families. Families with one immigrant parent were more likely to have low-income status than native families. After adjusting for age and low-income status, fathers in families with one immigrant parent had worser physical health than fathers in native families (indicated by the Charlson comorbidity index score with a regression coefficient of 0.05), especially with regard to cardiovascular diseases, cerebrovascular diseases, dementia, diabetes, renal diseases, tumors, and AIDS, with a range of adjusted odds ratios (aORs) from 1.13 to 1.45. Moreover, the risk of catastrophic illness in the fathers of families with one immigrant parent was higher than that in the fathers of native families. The fathers in families with one immigrant parent, in addition to poor physical health, had comparatively poor mental health, specifically with regard to ASD, major depressive disorder, bipolar disorder, and schizophrenia, with a range of aORs from 1.13 to 3.12. Further, the mortality rate in the fathers of families with one immigrant parent was higher than that in the fathers of native families (aOR 1.30, 95% CI 1.22-1.38).

**Table 1:** Sociodemographic variables and physical and mental health status of fathers and mothers in families with one immigrant parent and of those in native families.

Variable			Father							Mother				
			Families with one immigrant parent (n=90,670)		Native families (n=1,666,775)		Odds ratio^a^ (95% CI)		Families with one immigrant parent (n=91,270)		Native families (n=1,734,104)		Odds ratio^a^ (95% CI)	
**Sociodemographics^b^**														
	Age (years), mean (SD)	44.8 (7.4)		40.2 (0.8)		4.6 (4.5 to 4.6)			35.2 (5.1)		34.4 (6.0)	0.8 (0.7 to 0.9)		
	Low income, n (%)	9053 (9.98)		74,186 (4.45)		2.38 (2.33 to 2.44)			9108 (9.98)		86,163 (4.97)	2.12 (2. 07 to 2.17)		
**Geographical location in Taiwan, n (%)**														
	Northern	44,492 (49.07)		805,225 (48.31)		1.00			45,889 (50.28)		816,943 (47.11)	1.00		
	Central	19,313 (21.30)		418,668 (25.12)		0.83 (0.82 to 0.85)			20,598 (22.57)		444,424 (25.63)	0.83 (0.81 to 0.84)		
	Southern	23,683 (26.12)		404,455 (24.27)		1.06 (1.04 to 1.08)			22,748 (24.92)		428,076 (24.69)	0.94 (0.93 to 0.96)		
	Eastern	3183 (3.51)		38,427 (2.30)		1.50 (1.44 to 1.56)			2034 (2.23)		44,661 (2.58)	0.81 (0.77 to 0.85)		
**Physical disorders^c^**														
	Charlson comorbidity index, mean (SD)	1.1 (2.0)		0.8 (1.6)		0.05 (0.04 to 0.06)			0.2 (0.7)		0.6 (1.2)	–0.2 (–0.2 to –0.3)		
	Myocardial infarction, n (%)	828 (0.91)		6889 (0.41)		1.17 (1.04 to 1.27)			6 (0.01)		573 (0.03)	0.16 (0.06 to 0.42)		
	Congestive heart failure, n (%)	488 (0.54)		3201 (0.19)		1.45 (1.20 to 1.59)			55 (0.06)		2334 (0.13)	0.48 (0.34 to 0.62)		
	Peripheral vascular disease, n (%)	875 (0.97)		8291 (0.50)		1.28 (1.18 to 1.36)			148 (0.16)		6280 (0.36)	0.52 (0.42 to 0.59)		
	Cerebrovascular disease, n (%)	2889 (3.19)		23,924 (1.44)		1.27 (1.21 to 1.31)			246 (0.27)		12,261 (0.71)	0.41 (0.35 to 0.48)		
	Dementia, n (%)	1383 (1.53)		14,266 (0.86)		1.40 (1.31 to 1.51)			1010 (1.11)		34,136 (1.97)	0.57 (0.50 to 0.62)		
	Chronic pulmonary disease, n (%)	1807 (1.99)		13,902 (0.83)		0.97 (0.94 to 1.02)			121 (0.13)		6548 (0.38)	0.38 (0.36 to 0.40)		
	Connective tissue disease, n (%)	10,145 (11.19)		167,583 (10.05)		0.85 (0.80 to 0.97)			3782 (4.14)		188,174 (10.85)	0.34 (0.29 to 0.40)		
	Ulcer disease, n (%)	1045 (1.15)		17,395 (1.04)		0.95 (0.91 to 0.97)			651 (0.71)		39,232 (2.26)	0.56 (0.53 to 0.59)		
	Mild liver disease, n (%)	17,135 (18.90)		272,341 (16.34)		0.92 (0.89 to 0.97)			6865 (7.52)		241,908 (13.95)	0.49 (0.43 to 0.55)		
	Diabetes, n (%)	9905 (10.92)		87,297 (5.24)		1.36 (1.27 to 1.41)			834 (0.91)		43,403 (2.50)	0.42 (0.39 to 0.51)		
	Diabetes with end-organ damage, n (%)	2952 (3.26)		23,862 (1.43)		1.32 (1.25 to 1.39)			101 (0.11)		8777 (0.51)	0.24 (0.21 to 0.28)		
	Hemiplegia, n (%)	12,422 (13.70)		192,840 (11.57)		1.25 (1.16 to 1.32)			1725 (1.89)		80,778 (4.66)	0.42 (0.32 to 0.52)		
	Moderate or severe renal disease, n (%)	7182 (7.92)		121,505 (7.29)		1.20 (1.16 to 1.24)			1620 (1.77)		63,672 (3.67)	0.32 (0.30 to 0.34)		
	Any tumor, n (%)	3519 (3.88)		35,537 (2.13)		1.13 (1.06 to 1.24)			263 (0.29)		17,287 (1)	0.15 (0.13 to 0.17)		
	Leukemia, n (%)	1598 (1.76)		13,880 (0.83)		1.05 (0.80 to 1.31)			117 (0.13)		20,526 (1.18)	0.49 (0.30 to 0.70)		
	Lymphoma, n (%)	114 (0.13)		1455 (0.09)		0.92 (0.76 to 1.09)			30 (0.03)		1397 (0.08)	0.31 (0.20 to 0.45)		
	Moderate or severe liver disease, n (%)	133 (0.15)		1946 (0.12)		0.89 (0.85 to 0.92)			27 (0.03)		1831 (0.11)	0.59 (0.52 to 0.66)		
	Metastatic solid tumor, n (%)	743 (0.82)		6129 (0.37)		1.13 (1.02 to 1.25)			101 (0.11)		5596 (0.32)	0.45 (0.36 to 0.53)		
	AIDS, n (%)	70 (0.08)		648 (0.04)		1.38 (1.06 to 1.79)			22 (0.02)		476 (0.03)	0.70 (0.44 to 1.11)		
	Catastrophic illness, n (%)	5262 (5.80)		46,715 (2.80)		1.43 (1.40 to 1.45)			775 (0.85)		50,324 (2.90)	0.32 (0.30 to 0.34)		
**Mental disorders, n (%)^c^**														
	Autism spectrum disorder	10 (0.01)		97 (0.01)		2.73 (1.41 to 5.31)			3 (0.003)		91 (0.005)	0.18 (0.02 to 1.80)		
	Attention-deficit/hyperactivity disorder	57 (0.06)		2010 (0.12)		1.20 (0.90 to 1.56)			8 (0.01)		1564 (0.09)	0.08 (0.04 to 0.16)		
	Anxiety disorders	2027 (2.24)		33,331 (2)		0.87 (0.84 to 0.92)			880 (0.96)		44,898 (2.59)	0.38 (0.36 to 0.41)		
	Major depressive disorder	4345 (4.79)		59,754 (3.59)		1.13 (1.09 to 1.19)			1627 (1.78)		104,100 (6)	0.30 (0.28 to 0.32)		
	Bipolar disorder	918 (1.01)		10,496 (0.63)		1.42 (1.33 to 1.53)			236 (0.26)		16,740 (0.97)	0.26 (0.23 to 0.30)		
	Schizophrenia	1142 (1.26)		5403 (0.32)		3.12 (2.93 to 3.34)			182 (0.20)		6176 (0.36)	0.50 (0.44 to 0.55)		
Mortality, n (%)^b^			2608 (2.88)		18,907 (1.13)		1.30 (1.22 to 1.38)		33 (0.04)		6613 (0.38)		0.10 (0.06 to 0.15)	

^a^The values in this column could be odds ratio or the regression coefficient.

^b^Crude analysis was conducted without any adjustment.

^c^Analysis was adjusted for age, geographical location, and low-income status.

Conversely, mothers in families with one immigrant parent had better physical health (indicated by the Charlson comorbidity index score with an adjusted regression coefficient of –0.2), with lower risks in most physical disorders and catastrophic illnesses than mothers in native families (aOR range 0.15-0.57). Moreover, mothers in families with one immigrant parent had better mental health than mothers in native families, with a range of aORs from 0.08 to 0.50. Further, the mortality rate in mothers of families with one immigrant parent was lower than that in mothers of native families (aOR 0.10, 95% CI 0.06-0.15). We further restricted the time period of health care utilization after the delivery of the first child to make the time period in mothers of families with one immigrant parent and those of native families comparable, thereby resulting in a similar pattern of general health between the 2 groups (Table S2 of Multimedia Appendix 1).

We observed that the fathers had statistically poorer health with regard to most physical and mental disorders than the mothers, regardless of whether they belonged to native families or families with one immigrant parent. Furthermore, based on the moderation analysis, we found that such discrepancies in physical and mental disorders between fathers and mothers were more statistically profound in families with one immigrant parent, except for ASD (Table S3 of Multimedia Appendix 1).

Table 2 summarizes the sociodemographic data and the general health between the children of families with one immigrant parent and those of native families. The children of families with one immigrant parent had slightly better physical health than the children of native families (indicated by the Charlson comorbidity index score with an adjusted regression coefficient of –0.01), with a lower risk of cerebrovascular disease, chronic pulmonary disease, and connective tissue disease (aOR range 0.74-0.91) but a higher risk of leukemia (aOR 1.31) and liver diseases (aOR 1.24). A similar pattern was also found for mental health: children of families with one immigrant parent had comparatively lower risks of ADHD, CD/ODD, and anxiety disorders (aOR range 0.62-0.88). However, a higher risk of ASD was observed in the children of families with one immigrant parent than in the children of native families (aOR 1.13). In addition to general health, the adversities and accidents experienced by the children are summarized in Table 3. A higher risk of road traffic accidents was observed among children of families with one immigrant parent than among children of native families (aOR 1.11, 95% CI 1.07-1.16).

**Table 2:** Sociodemographic variables and physical and mental health of children of families with one immigrant parent and of those of native families.

Variable		Children of families with one immigrant parent (n=132,457)	Children of native families (n=2,637,191)	Odds ratio^a^ (95% CI)
**Sociodemographics^b^**				
	Sex (boys), n (%)	68,304 (51.57)	1,364,032 (51.72)	0.99 (0.98 to 1.00)
	Age (years), mean (SD)	6.6 (3.8)	6.7 (4.1)	0.1 (0.1 to 0.1)
	Low income, n (%)	14,152 (10.68)	147,543 (5.59)	2.02 (1.98 to 2.06)
**Geographical location in Taiwan, n (%)**				
	Northern	61,836 (46.68)	1,221,120 (46.30)	1.00
	Central	35,066 (26.47)	691,353 (26.22)	1.00 (0.99 to 1.02)
	Southern	32,918 (24.85)	666,032 (25.26)	0.98 (0.96 to 0.99)
	Eastern	2637 (1.99)	58,681 (2.23)	0.88 (0.85 to 0.92)
**Physical disorders^c^**				
	Charlson comorbidity index, mean (SD)	0.2 (0.5)	0.3 (0.5)	–0.01 (–0.01 to –0.01)
	Congestive heart failure, n (%)	159 (0.12)	3229 (0.12)	0.96 (0.81 to 1.18)
	Peripheral vascular disease, n (%)	25 (0.02)	363 (0.01)	1.32 (0.84 to 2.07)
	Cerebrovascular disease, n (%)	141 (0.11)	3302 (0.13)	0.82 (0.68 to 0.99)
	Chronic pulmonary disease, n (%)	70 (0.05)	1572 (0.06)	0.91 (0.88 to 0.95)
	Connective tissue disease, n (%)	25,652 (19.37)	593,158 (22.49)	0.74 (0.56 to 0.98)
	Ulcer disease, n (%)	61 (0.05)	2026 (0.08)	1.09 (0.93 to 1.26)
	Mild liver disease, n (%)	229 (0.17)	4605 (0.17)	1.23 (0.98 to 1.55)
	Diabetes, n (%)	63 (0.05)	1863 (0.07)	0.80 (0.61 to 1.05)
	Diabetes with end-organ damage, n (%)	5 (0.004)	205 (0.01)	0.66 (0.26 to 1.64)
	Hemiplegia, n (%)	96 (0.07)	1842 (0.07)	0.83 (0.63 to 1.09)
	Moderate or severe renal disease, n (%)	352 (0.27)	6073 (0.23)	0.96 (0.84 to 1.10)
	Any tumor, n (%)	233 (0.18)	5373 (0.20)	1.11 (0.85 to 1.40)
	Leukemia, n (%)	78 (0.06)	1499 (0.06)	1.31 (1.02 to 1.73)
	Lymphoma, n (%)	76 (0.06)	1220 (0.05)	1.33 (0.87 to 1.96)
	Moderate or severe liver disease, n (%)	32 (0.02)	485 (0.02)	1.24 (1.11 to 1.39)
	Metastatic solid tumor, n (%)	27 (0.02)	453 (0.02)	1.33 (0.87 to 2.02)
	AIDS, n (%)	3 (0.002)	51 (0.002)	0.74 (0.08 to 5.81)
	Catastrophic illness	1690 (1.28)	36,746 (1.39)	0.96 (0.89 to 1.01)
**Mental disorders, n (%)^d^**				
	Autism spectrum disorder	1063 (0.80)	20,251 (0.77)	1.13 (1.03 to 1.20)
	Attention-deficit/hyperactivity disorder	3665 (2.77)	90,698 (3.44)	0.88 (0.84 to 0.91)
	Tic disorder	440 (0.33)	11,201 (0.43)	0.89 (0.80 to 1.05)
	Conduct disorder/oppositional defiant disorder	219 (0.33)	6268 (0.24)	0.77 (0.68 to 0.89)
	Anxiety disorders	26 (0.17)	929 (0.04)	0.62 (0.40 to 0.96)
	Major depressive disorder	24 (0.02)	833 (0.03)	0.70 (0.46 to 1.09)
	Bipolar disorder	5 (0.004)	317 (0.01)	0.44 (0.19 to 1.05)
	Schizophrenia	3 (0.002)	59 (0.002)	0.03 (0.72 to 2.79)
Mortality, n (%)^b^		684 (0.52)	14,389 (0.55)	1.06 (0.97 to 1.20)

^a^The values in this column could be odds ratio or the regression coefficient.

^b^Crude analysis was conducted without any adjustment.

^c^Analysis was adjusted for child year of birth, geographical location, sex, parental age, low-income status, and physical disorder status (ie, Charlson comorbidity index).

^d^Analysis was adjusted for child year of birth, geographical location, sex, parental age, low-income status, and mental disorder status (ie, autism spectrum disorder, attention deficit and hyperactivity disorder, and schizophrenia).

**Table 3:** Child adversity and accidents reported among children of families with one immigrant parent and among those of native families.^a^

Variable	Children of families with one immigrant parent (n=132,457), n (%)	Children of native families (n=2,637,191), n (%)	Adjusted odds ratio (95% CI)
Domestic violence	117 (0.09)	1913 (0.07)	1.08 (0.90-1.31)
Maltreatment	844 (0.64)	20,044 (0.76)	0.62 (0.59-0.70)
Sexual assault survivors	213 (0.16)	3675 (0.14)	1.00 (0.87-1.19)
Road traffic accident	2690 (2.03)	48,589 (1.84)	1.11 (1.07-1.16)

^a^Analysis was adjusted for child year of birth, geographical location, sex, and low-income status.

## Discussion

### Principal Findings

This study provides a comprehensive view of the general health among members of families with one immigrant parent and among those of native families in Taiwan. Specifically, we found that the fathers in families with one immigrant parent had a higher mortality rate and poorer physical and mental health than the fathers in native families. Conversely, the mothers in families with one immigrant parent had lower mortality rates and better physical and mental health than the mothers in native families. Similarly, the children in families with one immigrant parent showed slightly better physical and mental health than the children in native families. As per our findings, poorer general health in fathers of families with one immigrant parent should be considered as an important public health issue.

### Methodological Considerations

Some methodological considerations need to be mentioned before further discussion. First, there is some diversity in the immigrant families in Taiwan; although most immigrant families are formed through international marriage brokerage agencies in Taiwan, not all families adopt this approach. Families formed by love marriage may have better health than those formed through brokerage because strong bonds between couples have positive impacts on physical and mental health [42]. Second, the medical data of the mothers and children of families with one immigrant parent may not reflect their real health status, because the medical information of these immigrant mothers before their immigration was not collected in our national registered database. Moreover, although we controlled for the time issues by restricting the period to after the delivery of their first child to address the lack of data for married immigrants, some medical barriers such as language difficulties [43], inadequate health literacy [44], and inconvenient access to health care institutions [45] may lead to lower utilization of health care services. Furthermore, because mothers are the main caregivers of children in Taiwan, the lower utilization of health care services is also expected to extend to the children of families with one immigrant parent. Third, some immigrant mothers without legal residence permits may not be covered in the national registered birth data set. Fourth, the age ranges of our sample were limited because the national registered birth data sets were established from 2004 to 2016. Therefore, the medical data may not cover common diseases in subsequent adulthood. Finally, the medical data of individuals in Taiwan may differ from those in other countries that have a private health insurance system rather than a national health insurance system, and some information such as education level, employment status, and marital status (eg, divorce or separation) is not fully available in our databases.

With regard to the general health of the fathers in families with one immigrant parent, we found that fathers were a special subpopulation with health vulnerability; they had higher mortality and morbidity due to various physical and mental disorders, comprising cardiovascular diseases, cerebrovascular diseases, dementia, diabetes, renal diseases, tumors, AIDS, ASD, major depressive disorder, bipolar disorder, and schizophrenia, after adjusting for sociodemographic variables. Low SES may be the main reason for explaining their health vulnerability [46,47], and some studies have also reported low SES in fathers of families with one immigrant parent. Low education levels [24,25] have been reported to be associated with poor physical and mental health [48,49], which may be further mediated by inadequate health literacy and health promotion behaviors [50,51]. Moreover, most of the fathers in families with one immigrant parent were low-skilled laborers [26], and low-skilled labor has been reported to lead to a shorter life expectancy [52] and higher health risks [53] due to harmful work styles, the lack of health promotion behaviors, and unhealthy living habits (eg, no regular exercise, smoking, poor diet) [53]. Furthermore, education and occupation are correlated with each other [54]. Our findings for AIDS was in line with a study on the relationship between SES and AIDS [55], which also emphasized the neglect of AIDS prevention in the heterosexual population of Taiwan. As a result, for fathers of families with one immigrant parent, both low SES and poor health [56] made it difficult to find suitable partners among native women, and marriage brokerage agencies thus became another option to get married.

We observed that mothers in families with one immigrant parent were generally healthier than mothers in native families. The healthy immigrant effect is a possible reason to explain our finding, where healthy people have more advantages to migrate abroad. The salmon bias effect may be less likely to contribute to explaining our findings because the salmon bias effect has been reported more profoundly in mortality cases and in older people [9]. However, we observed comprehensive differences in terms of physical and mental health and mortality in the mothers of families with one immigrant parent, and these mothers were generally middle-aged adults. Most likely, the health differences in terms of physical and mental health and mortality between the mothers in families with one immigrant parent and the mothers in native families can be explained by the healthy immigrant effect, and the health differences may be slightly overestimated because of the salmon bias effect. It is worth mentioning that some evidence was reported in line with the healthy immigrant effect in Taiwan. Married immigrants were observed to have better mental health [12], including lower risks of major depressive disorder [11] and postpartum depression [13], after adjusting for sociodemographic variables, whereas some findings were not in line with the healthy immigrant effect, and the inconsistent findings may be a result of the differences in methodology, culture, and sample characteristics. For studies with contradictory findings in Taiwan, the authors overlooked common confounding effects (eg, sociodemographic variables) between immigrant status and mental health [16,18,57]. In Korea, married immigrants were observed to have poor mental health after accounting for the sociodemographic variables [17,19]. The different findings between Taiwan and Korea may be due to the difference in the xenophobic atmosphere [58-60] or the relative health status in the native populations [61]. Furthermore, the healthy immigrant effect was found to disappear gradually after immigration [62].

We found that the children of families with one immigrant parent generally had slightly better health than the children of native families, but they had higher risks for some diseases (ie, leukemia, liver diseases, and ASD) and road traffic accidents. Parental health and the sociodemographic status may explain these risks. The better health of the mothers in families with one immigrant parent may explain the better health of the children [63]. In contrast, poor paternal SES may adversely affect the health of children [64]. Furthermore, we found that both the fathers and children of families with one immigrant parent had higher risks of ASD, suggesting the importance of genetic heritability. Finally, the children of families with one immigrant parent had higher risks of accidents, which may be explained by the usage of different types of vehicles. Motorcycles, rather than passenger cars, may be a more affordable choice for families with one immigrant parent because the cost of transportation is much lower; however, motorcycles have been reported to lead to a higher risk of traffic accidents than passenger cars [65].

### Conclusions

Our study is the first national cohort study, to the best of our knowledge, to comprehensively elucidate the health status of families with one immigrant parent in Taiwan with substantial evidence, and our findings indicate that family health is nonhomogeneous within such families. We found that fathers in families with one immigrant parent generally had a poor physical and mental health but not the mothers. Moreover, the children of families with one immigrant parent generally had slightly better general health than the children of native families, but they had higher risks for some diseases (ie, leukemia, liver diseases, and ASD) and road traffic accidents. These results indicate that since there are health inequalities within the members of families with one immigrant parent, they should be provided with adequate prenatal care and parenting education.

## Appendix

Multimedia Appendix 1 Supplementary tables and methods.

## Abbreviations

ADHD: attention deficit and hyperactivity disorder
aOR: adjusted odds ratio
ASD: autism spectrum disorder
CD/ODD: conduct disorder or oppositional defiant disorder
SES: socioeconomic status
