# Supplementary material for: Comprehensive Comparisons of Family Health Between Families With One Immigrant Parent and Native Families in Taiwan: Nationwide Population-Based Cohort Study
Source: JMIR Public Health Surveill. 2022 Dec 19;8(12):e33624. doi: 10.2196/33624 (PMC9808584; doi:10.2196/33624)
Supplement: Multimedia Appendix 1 [file publichealth_v8i12e33624_app1.docx]

Supplementary material (Online-only)

**List:**

**Supplementary Methods**

Table S1. The categorical of diseases for catastrophic illnesses in Taiwan

Table S2. Sociodemographics and physical and mental health between mothers of transnational marriage immigrant families and native families after the delivery of first child

Table S3. Sociodemographics and physical and mental health between fathers and mothers in international marriage immigrant families and native families

# Supplementary Method

**Used datasets**

*Cause of Death Data*

National cause of death data is managed by the Office of Statistics, Department of Health, which has been available in electronic form since 1971. It contains the identity of dead people, death date and their cause of death, using the International Classification of Diseases classification system [1].

*Registry for Catastrophic Illness Patients*

Taiwan’s National Health Insurance system has defined catastrophic illnesses for reducing economic burden and protecting the medical right with regards to access to necessary medical care in the enrollees with catastrophic illnesses, listed in Supplementary Table S1, in the National Health Insurance programs [2]. Registry for Catastrophic Illness Patients is established by extracting any individuals who matched the co-payment related catastrophic illness code in the National Health Insurance Research Database.

*Family Violence Data and Reported Data of Sexual Assault*

Family Violence Data, Reported Data of Protection of Child and Youths, and Reported Data of Sexual Assault are managed by Taiwan’s Ministry of Health and Welfare with the insurance of privacy and safety of data [3]. For Family Violence Data, it contains information about the identity of the child and youth victims and perpetrators of families experiencing family violence, the date of family violence. For Reported Data of Protection of Child and Youths, it contains the information of the children and youths who are involved in the maltreatment, the use of illicit substances, and crimes of assault and battery. We used the Reported Data of Protection of Child and Youths to identify whether our child participants had the maltreatment. Finally, for Reported Data of Sexual Assault, it contains the information of the identity of the child and youth victims and perpetrators of sexual assault and their relationships.

*Traffic Accident Data*

Traffic Accident Data is recorded and maintained by the Ministry of Transportation and Communications, and is released to researchers to investigate factors related to traffic accidents since 2003 [3]. It contains information about accident frequency and fatality or injury for traffic accidents and the identity of drivers and passengers.

**References**

**1.** Lu T-H, Lee M-C, Chou M-C. Accuracy of cause-of-death coding in Taiwan: Types of miscoding and effects on mortality statistics. International Journal of Epidemiology*.* 2000;29(2):336-343. [doi: 10.1093/ije/29.2.336] [Medline: 10817134]

**2.** Nan-Ping Y, Yi-Hui L, Chi-Yu C, Jin-Chyr H, I-Liang Y, Nien-Tzu C, et al. Comparisons of medical utilizations and categorical diagnoses of emergency visits between the elderly with catastrophic illness certificates and those without. BMC Health Services Research*.* 2013;13(1):152. [doi: 10.1186/1472-6963-13-152] [Medline: 23622501]

**3.** Lin L-Y, Warren-Gash C, Smeeth L, Chen P-C. Data resource profile: The national health insurance research database (NHIRD). Epidemiology and Health*.* 2018;40:e2018062. [doi: 10.4178/epih.e2018062] [Medline: 30727703]

**Table S1. The categorical of diseases for catastrophic illnesses in Taiwan**

| Category of disease | |
| --- | --- |
|  | Cancer |
|  | Homological abnormality |
|  | Renal failure |
|  | Generalized autoimmune diseases |
|  | Persisted autoimmune disorders |
|  | Chronic mental disorders |
|  | Congenital metabolic disorders |
|  | Major organs abnormality |
|  | Massive burns |
|  | Major organs transplantation |
|  | Complicated nervous, musculoskeletal disorders |
|  | Injury severity score more than 16 |
|  | Respiratory failure |
|  | Un-corrected mal-nutrition status |
|  | Persisted complications due to decompression sickness or air embolism |
|  | Myasthenia gravis |
|  | Congenital immune deficiency |
|  | Spinal cord injuries |
|  | Occupational diseases |
|  | Cerebro-vascular diseases (acute stage |
|  | Multiple sclerosis |
|  | Congenital muscular dystrophy |
|  | Congenital anomalies integument |
|  | Leprosy |
|  | Complicated liver cirrhosis |
|  | Co-morbidity due to premature birth |
|  | Toxic effect of arsenic and its compounds |
|  | Motor neuron disease |
|  | Creutzfeldt-jakob disease |
|  | Other rare diseases |

**Table S2. Sociodemographic variables and physical and mental health of children of families with one immigrant parent and those of native families after the delivery of first child**

|  | Mother | |  |
| --- | --- | --- | --- |
|  | Families with one immigrant parent | Native families | OR (95% CI)/ B (95% CI) |
| Variable | N=91270 | N=1734104 |  |
| Sociodemographics^a^ |  |  |  |
| Age, Mean (SD) | 35.2 (5.1) | 34.4 (6.0) | 0.8 (0.7 to 0.9) |
| Low-income, N (%) | 9108 (0.100) | 86163 (0.050) | 2.12 (2. 07 to 2.17) |
| Physical Disorders^b^ |  |  |  |
| Myocardial infarct, N (%) | 6 (<0.001) | 494 (<0.001) | 0.13 (0.05 to 0.34) |
| Congestive heart failure, N (%) | 55 (0.001) | 2072 (0.001) | 0.59 (0.47 to 0.73) |
| Peripheral vascular disease, N (%) | 148 (0.002) | 4100 (0.002) | 0.80 (0.69 to 0.92) |
| Cerebrovascular disease, N (%) | 246 (0.003) | 9433 (0.005) | 0.59 (0.53 to 0.66) |
| Dementia, N (%) | 1010 (0.011) | 27288 (0.016) | 0.76 (0.72 to 0.80) |
| Chronic pulmonary disease, N (%) | 3782 (0.041) | 149998 (0.086) | 0.53 (0.52 to 0.55) |
| Connective tissue disease, N (%) | 651 (0.007) | 29165 (0.017) | 0.53 (0.49 to 0.56) |
| Ulcer disease, N (%) | 6865 (0.075) | 168751 (0.097) | 0.88 (0.86 to 0.90) |
| Mild liver disease, N (%) | 1725 (0.019) | 57622 (0.033) | 0.73 (0.70 to 0.76) |
| Diabetes, N (%) | 834 (0.009) | 39873 (0.023) | 0.51 (0.48 to 0.54) |
| Diabetes with end organ damage, N (%) | 101 (0.001) | 8528 (0.005) | 0.27 (0.23 to 0.32) |
| Hemiplegia, N (%) | 121 (0.001) | 5159 (0.003) | 0.54 (0.46 to 0.63) |
| Moderate or severe renal disease, N (%) | 263 (0.003) | 13168 (0.008) | 0.44 (0.40 to 0.49) |
| Any tumor, N (%) | 117 (0.001) | 20526 (0.012) | 0.14 (0.12 to 0.17) |
| Leukemia, N (%) | 30 (<0.001) | 1294 (0.001) | 0.48 (0.35 to 0.66) |
| Lymphoma, N (%) | 27 (<0.001) | 1593 (0.001) | 0.32 (0.23 to 0.46) |
| Moderate or severe liver disease, N (%) | 1620 (0.018) | 51403 (0.030) | 0.76 (0.72 to 0.79) |
| Metastatic solid tumor, N (%) | 101 (0.001) | 4930 (0.003) | 0.58 (0.49 to 0.69) |
| Acquired immunodeficiency syndrome, N (%)^c^ | 22 (<0.001) | 448 (<0.001) | 0.67 (0.45 to 0.98) |
| Charlson Comorbidity Index, Mean (SD) | 0.2 (0.7) | 0.4 (1.1) | -0.13 (-0.12 to -0.13) |
| Catastrophic illness, N (%) | 775 (0.008) | 33340 (0.019) | 0.56 (0.53 to 0.59) |
| Mental disorders^b^ |  |  |  |
| Autism spectrum disorder, N (%) | 3 (<0.001) | 83 (0.005) | 0.19 (0.01 to 1.84) |
| Attention-deficit/hyperactivity disorder, N (%)^c^ | 8 (<0.001) | 631 (<0.001) | 0.26 (0.15 to 0.46) |
| Anxiety disorders, N (%) | 880 (0.010) | 36798 (0.021) | 0.49 (0.46 to 0.52) |
| Major depressive disorder, N (%) | 1627 (0.018) | 73098 (0.042) | 0.42 (0.40 to 0.44) |
| Bipolar disorder, N (%) | 236 (0.003) | 12799 (0.007) | 0.33 (0.29 to 0.37) |
| Schizophrenia, N (%) | 182 (0.002) | 4764 (0.003) | 0.68 (0.60 to 0.76) |
| Mortality, N (%)^a^ | 33 (<0.001) | 6600 (0.004) | 0.11 (0.08 to 0.15) |

B = regression coefficient, OR = odds ratio, CI = confidence interval SD = standard deviation

^a^ Crude analysis was conducted without any adjustment

^b^Analysis was adjusted for age and low-income status.

^c^Abbreviations: AIDS = acquired immunodeficiency syndrome; ADHD = attention deficit and hyperkinetic disorders.

Table S3. Sociodemographics and physical and mental health between fathers and mothers in families with one immigrant parent and and native families

|  | Families with one immigrant parent | |  | Native families | |  | Moderation analysis of father-mother difference |
| --- | --- | --- | --- | --- | --- | --- | --- |
|  |  |  |  |  |  |  | Families with one immigrant parent  vs.  native families |
|  | Father | Mother | OR (95% CI)/B (95% CI) | Native families | Mother | OR (95% CI)/B (95% CI) | OR (95% CI)/B (95% CI) |
| Variable | N=90670 | N=91270 |  | N=1666775 | N=1734104 |  |  |
| **Physical Disorders** |  |  |  |  |  |  |  |
| Charlson comorbidity index, Mean (SD) | 1.1 (2.0) | 0.2 (0.7) | 0.9 (0.9 to 0.9) | 0.8 (1.6) | 0.6 (1.2) | 0.2 (0.2 to 0.2) | 0.7 (0.7 to 0.7) |
| Myocardial infarction, n (%) | 828 (0.91) | 6 (0.01) | 140.18 (62.79 to 312.96) | 6889 (0.41) | 573 (0.03) | 12.56 (11.53 to 13.67) | 11.16 (4.98 to 25.03) |
| Congestive heart failure, n (%) | 488 (0.54) | 55 (0.06) | 8.97 (6.79 to 11.86) | 3201 (0.19) | 2334 (0.13) | 1.43 (1.35 to 1.51) | 6.27 (4.72 to 8.34) |
| Peripheral vascular disease, n (%) | 875 (0.97) | 148 (0.16) | 6.00 (5.04 to 7.14) | 8291 (0.50) | 6280 (0.36) | 1.38 (1.33 to 1.42) | 4.35 (3.64 to 5.20) |
| Cerebrovascular disease, n (%) | 2889 (3.19) | 246 (0.27) | 12.18 (10.69 to 13.88) | 23924 (1.44) | 12261 (0.71) | 2.05 (2.00 to 2.09) | 5.94 (5.20 to 6.79) |
| Dementia, n (%) | 1383 (1.53) | 1010 (1.11) | 1.38 (1.28 to 1.50) | 14266 (0.86) | 34136 (1.97) | 0.43 (0.42 to 0.44) | 3.21 (2.97 to 3.47) |
| Chronic pulmonary disease, n (%) | 1807 (1.99) | 121 (0.13) | 15.32 (12.74 to 18.42) | 13902 (0.83) | 6548 (0.38) | 2.22 (2.15 to 2.29) | 6.90 (5.72 to 8.32) |
| Connective tissue disease, n (%) | 10145 (11.19) | 3782 (4.14) | 2.91 (2.80 to 3.03) | 167583 (10.05) | 188174 (10.85) | 0.92 (0.91 to 0.92) | 3.16 (3.04 to 3.29) |
| Ulcer disease, n (%) | 1045 (1.15) | 651 (0.71) | 1.62 (1.47 to 1.79) | 17395 (1.04) | 39232 (2.26) | 0.46 (0.45 to 0.46) | 3.52 (3.19 to 3.89) |
| Mild liver disease, n (%) | 17135 (18.90) | 6865 (7.52) | 2.86 (2.78 to 2.95) | 272341 (16.34) | 241908 (13.95) | 1.20 (1.20 to 1.21) | 2.38 (2.32 to 2.45) |
| Diabetes, n (%) | 9905 (10.92) | 834 (0.91) | 13.30 (12.38 to 14.28) | 87297 (5.24) | 43403 (2.50) | 2.15 (2.13 to 2.18) | 6.19 (5.75 to 6.65) |
| Diabetes with end-organ damage, n (%) | 2952 (3.26) | 101 (0.11) | 30.38 (24.91 to 37.05) | 23862 (1.43) | 8777 (0.51) | 2.86 (2.79 to 2.93) | 10.62 (8.70 to 12.97) |
| Hemiplegia, n (%) | 12422 (13.70) | 1725 (1.89) | 8.24 (7.83 to 8.67) | 192840 (11.57) | 80778 (4.66) | 2.68 (2.66 to 2.70) | 3.07 (2.92 to 3.24) |
| Moderate or severe renal disease, n (%) | 7182 (7.92) | 1620 (1.77) | 4.76 (4.51 to 5.03) | 121505 (7.29) | 63672 (3.67) | 2.06 (2.04 to 2.08) | 2.31 (2.19 to 2.44) |
| Any tumor, n (%) | 3519 (3.88) | 263 (0.29) | 13.97 (12.32 to 15.84) | 35537 (2.13) | 17287 (1.00) | 2.16 (2.12 to 2.20) | 6.47 (5.70 to 7.34) |
| Leukemia, n (%) | 1598 (1.76) | 117 (0.13) | 13.98 (11.58 to 16.87) | 13880 (0.83) | 20526 (1.18) | 0.70 (0.69 to 0.72) | 19.97 (16.53 to 24.12) |
| Lymphoma, n (%) | 114 (0.13) | 30 (0.03) | 3.83 (2.56 to 5.72) | 1455 (0.09) | 1397 (0.08) | 1.08 (1.01 to 1.17) | 3.55 (2.36 to 5.34) |
| Moderate or severe liver disease, n (%) | 133 (0.15) | 27 (0.03) | 4.96 (3.28 to 7.51) | 1946 (0.12) | 1831 (0.11) | 1.11 (1.04 to 1.18) | 4.47 (2.94 to 6.79) |
| Metastatic solid tumor, n (%) | 743 (0.82) | 101 (0.11) | 7.46 (6.06 to 9.18) | 6129 (0.37) | 5596 (0.32) | 1.14 (1.10 to 1.18) | 6.54 (5.30 to 8.08) |
| Acquired immunodeficiency syndrome, n (%) | 70 (0.08) | 22 (0.02) | 3.20 (1.98 to 5.17) | 648 (0.04) | 476 (0.03) | 1.42 (1.26 to 1.59) | 2.25 (1.37 to 3.70) |
| Catastrophic illness, n (%) | 5262 (5.80) | 775 (0.85) | 7.19 (6.67 to 7.76) | 46715 (2.80) | 50324 (2.90) | 0.96 (0.95 to 0.98) | 7.49 (6.94 to 8.08) |
| **Mental disorders** |  |  |  |  |  |  |  |
| Autistic spectrum disorder, n (%) | 10 (0.01) | 3 (0.003) | 3.36 (0.92 to 12.19) | 97 (0.01) | 91 (0.005) | 1.11 (0.83 to 1.48) | 3.03 (0.80 to 11.42) |
| Attention-deficit/hyperactivity disorder, n (%) | 57 (0.06) | 8 (0.01) | 7.18 (3.42 to 15.04) | 2010 (0.12) | 1564 (0.09) | 1.34 (1.25 to 1.43) | 5.36 (2.54 to 11.29) |
| Anxiety disorders, n (%) | 2027 (2.24) | 880 (0.96) | 2.35 (2.17 to 2.54) | 33331 (2.00) | 44898 (2.59) | 0.77 (0.76 to 0.78) | 3.05 (2.82 to 3.31) |
| Major depressive disorder, n (%) | 4345 (4.79) | 1627 (1.78) | 2.77 (2.62 to 2.94) | 59754 (3.59) | 104100 (6.00) | 0.58 (0.58 to 0.59) | 4.78 (4.52 to 5.05) |
| Bipolar disorder, n (%) | 918 (1.01) | 236 (0.26) | 3.95 (3.42 to 4.55) | 10496 (0.63) | 16740 (0.97) | 0.65 (0.63 to 0.67) | 6.08 (5.24 to 7.04) |
| Schizophrenia, n (%) | 1142 (1.26) | 182 (0.20) | 6.38 (5.46 to 7.47) | 5403 (0.32) | 6176 (0.36) | 0.91 (0.88 to 0.94) | 7.01 (5.98 to 8.22) |
| Mortality, n (%) | 2608 (2.88) | 33 (0.04) | 81.88 (58.08 to 115.44) | 18907 (1.13) | 6613 (0.38) | 3.00 (2.91 to 3.08) | 27.29 (19.33 to 38.53) |

B = regression coefficient, OR = odds ratio, CI = confidence interval SD = standard deviation.

For the moderation analysis, the native families servers as the reference group.
